# SARS-CoV-2 Variability in Patients and Wastewaters—Potential Immuno-Modulation during the Shift from Delta to Omicron

**DOI:** 10.3390/biomedicines10092080

**Published:** 2022-08-25

**Authors:** Ahlam Chaqroun, Cédric Hartard, Thomas Josse, Audrey Taverniers, Hélène Jeulin, Christophe Gantzer, John M. Murray, Obepine Consortium, Isabelle Bertrand, Evelyne Schvoerer

**Affiliations:** 1 CNRS, LCPME, Université de Lorraine, F-54000 Nancy, France; 2 Laboratoire de Virologie-Microbiologie, Hôpital Universitaire de Nancy, Université de Lorraine, F-54000 Nancy, France; 3 School of Mathematics and Statistics, UNSW Sydney, Sydney, NSW 2052, Australia

**Keywords:** SARS-CoV-2 variability, shift, Delta, Omicron, immune modulation

## Abstract

The continuous emergence of SARS-CoV-2 variants favors potential co-infections and/or viral mutation events, leading to possible new biological properties. The aim of this work was to characterize SARS-CoV-2 genetic variability during the Delta–Omicron shift in patients and in a neighboring wastewater treatment plant (WWTP) in the same urban area. The surveillance of SARS-CoV-2 was performed by routine screening of positive samples by single nucleotide polymorphism analysis within the S gene. Moreover, additionally to national systematic whole genome sequencing (WGS) once a week in SARS-CoV-2-positive patients, WGS was also applied when mutational profiles were difficult to interpret by routine screening. Thus, WGS was performed on 414 respiratory samples and on four wastewater samples, northeastern France. This allowed us to report (i) the temporally concordant Delta to Omicron viral shift in patients and wastewaters; (ii) the characterization of 21J (Delta) and 21K (Omicron)/BA.1-21L (Omicron)/BA.2-BA.4 mixtures from humans or environmental samples; (iii) the mapping of composite mutations and the predicted impact on immune properties in the viral Spike protein.

## 1. Introduction

The genetic mutation rate of Severe Acute Respiratory Syndrome Coronavirus 2 (SARS-CoV-2) has been widely discussed and recently estimated at a rate of approximately 1.2 × 10^−3^ per site per year [1]. Indeed, at the beginning of the viral emergence, a possible active proofreading repair able to maintain genome and viral fitness was considered to favor genomic RNA genome conservation. However, during the explosive pandemic, a strong SARS-CoV-2 diversification became evident, leading to a huge worry about host-related antiviral immunity, either natural or vaccine-induced protection. This SARS-CoV-2 genomic variability can induce amino acid changes, in particular in the Spike glycoprotein (encoded by S gene), involved in the attachment to the human Angiotensin-Converting Enzyme 2 (ACE2) receptor and other putative receptors, therefore, favoring the continuous emergence of variants. In addition, the co-circulation of several mutant strains increases the risk of co-infection, which may modify the viral pathogeny and eventually promote potential inter-strain recombination [1,2], mainly occurring in ORF1a and in the S gene [3], especially in the 174–2692 nt region of ORF1ab, 21,801–22,281 nt and 24,174–24,648 nt in the S gene in Sarbecoviruses [3,4]. However, the literature on co-infection by different SARS-CoV-2 variants and potential subsequent changes in the biological properties of this pandemic virus remains limited [3,4,5]. A recent work in France by P Combes at al showed mutation profiles suggesting that a Delta/Omicron population mixture was observed in 7 nasopharyngeal swabs out of 3831 respiratory samples in a high co-circulation period of the two variants. Co-infections can further lead to genetic recombination, observed in 1 of the 3831 samples, the authors suggesting that such chimeric variants with unpredictable epidemic or pathogenic properties might regularly occur [5].

Thus, due to the co-circulation of various SARS-CoV-2 strains within populations and in infected individual subjects, new variants with advantageous mutations and/or composite mutations can emerge. Subsequent new viral variants might carry new biological properties, such as viral escape from host-related immune response [6], specific human tissue or animal tropism [7], and possible modulations in SARS-CoV-2 properties in the environment through the Spike protein influencing viral behavior in samples, such as wastewaters [8].

In this context, additionally to the detection and sequencing of SARS-CoV-2 strains in patients, a promising approach to monitor the viral spreading is wastewater-based epidemiology (WBE) because of viral fecal shedding from infected patients. The exploration of viral genetic variability in human samples has been considered as a key tool by health workers, while WBE could allow us to take into account not only the symptomatic patients but also the asymptomatic infected people for a global survey of SARS-CoV-2 genomic evolution [8].

The aim of this work was to study SARS-CoV-2 genetic variability during the shift from Delta to Omicron waves in patients’ respiratory samples and in four wastewater samples, northeastern France. This was undertaken to investigate overall viral genetic evolution during the co-circulation of different SARS-CoV-2 variants and, for a few variant mixtures, to explore, by bioinformatics, modulations within antigenic host spots of the viral Spike protein.

## 2. Materials and Methods

### 2.1. Clinical Samples

From December 2021 to April 2022, nasopharyngeal swabs were evaluated for SARS-CoV-2 by real-time Reverse Transcription Polymerase Chain Reaction (RT-qPCR) targeting the RNA-dependent RNA polymerase (RdRp) genes IP2 and IP4, in the context of initial diagnosis of COVID-19-related symptoms in patients received in the university hospital of Nancy, France [9]. The surveillance of SARS-CoV-2 was performed by prospective screening of positive samples by single nucleotide polymorphism analysis within S gene (L452R/K417N). The viral sequencing was conducted locally in the hospital as part of the national SARS-CoV-2 molecular surveillance. Additionally to national systematic whole genome sequencing (WGS) once a week, WGS was also realized when mutational profiles were difficult to interpret on routine screening within S gene.

According to the French sanitary authorities, samples were PCR screened, among which around 10% were ultra-deeply sequenced (Deepcheck Whole Genome SARS-CoV-2 genotyping V3–V4, ABL SA Group, Metz, France), based on the nCoV-2019 sequencing protocol v3 (LoCost)-ARTIC network [10]. SARS-CoV-2 temporal genetic variability in general population followed-up at the university hospital was explored on 414 samples according to Pangolin/Nextrain classification at a mutation threshold above 20% to characterize major variants. Potential mixtures of variants of concern (VOCs) Delta and Omicron were also evaluated at a mutation threshold of 5% covering major and minor variants; then variant proportions in each mixture were determined according to covariants, CoV-Spectrum data, Nextclade assignment [11] and alignment with Wuhan reference strain (EPI ISL 402124), Delta (EPI ISL 11448607), and Omicron sequences (EPI_ISL_11352845; EPI_ISL_12179603) from GISAID (Global Initiative on Sharing Avian Influenza Data, http://gisaid.org, assessed on 1 March 2022). Analyses were also performed with the Recombination Detection Program software (RDP v4.5) [12] on the human samples showing SARS-CoV-2 variants mixtures, to investigate for possible viral recombinants by using RDP [13], GENECONV [14], BootScan [15], MaxChi [16], Chimaera [17], SiScan [18], and 3Seq programs [19].

To explore potential immune modulation that could be generated by SARS-CoV-2 variant mixtures, the hydrophilicity prediction was realized on Delta variant receptor-binding domain, on a putative Delta variant harboring various mutations in receptor-binding domain (RBD), with the Wuhan viral strain as a reference. The SARS-CoV-2 RBD was analyzed for the hydrophilicity correlated with the immunogenicity by using a free epitope database and prediction resource—IEDB (Immune Epitope Database, www.iedb.org, assessed on 1 March 2022) [20].

### 2.2. Wastewater Samples

Four wastewater samples (WW) collected in Nancy, France in 2022 (ww1—January 16; ww2—February 16; ww3—March 13; ww4—April 24) were also sequenced for the whole-SARS-CoV-2 genome and the sequences were submitted in GenBank (Table 1).

The WW samples were first concentrated from a volume of 100–200 mL to a volume of 5 mL using Centricon Plus 70 (100 kD) ultrafiltration, then purified using phenol-chloroform and extracted using the NucliSENS^®^easyMAG™ (bioMérieux, Marcy L’Etoile, France). A secondary concentration was carried out using RNA Clean & Concentrator kit (Zymo-Research, Lucerna, Switzerland) to final volume of 15 to 20 µL. Based on a WW sequencing procedure by WGS Ifremer protocol, an optimization of amplicon preparation was carried out with modification of conditions to the ABL procedure (Whole Genome SARS-CoV-2 genotyping V3-4, ABL SA Group, Metz, France) to allow us to explore the potential composite assignation of SARS-CoV-2 sequences from the water treatment plant [21].

## 3. Results

### 3.1. Temporal Variability and Mixtures of Variants in Nasopharyngeal Samples

**Figure 1:**
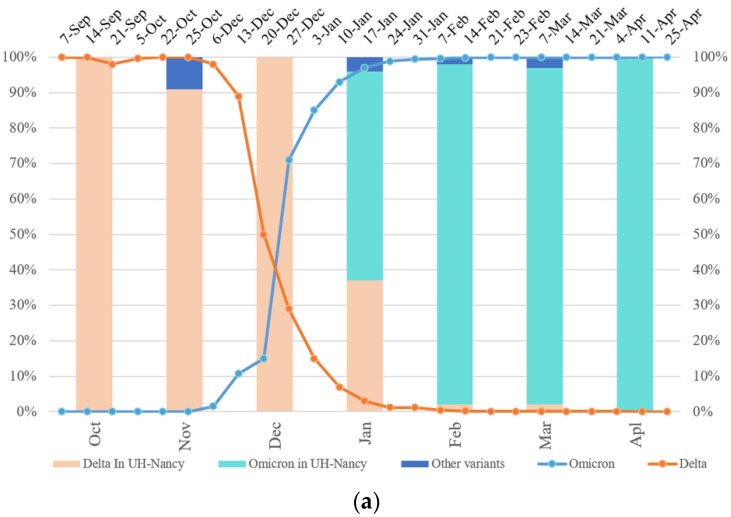

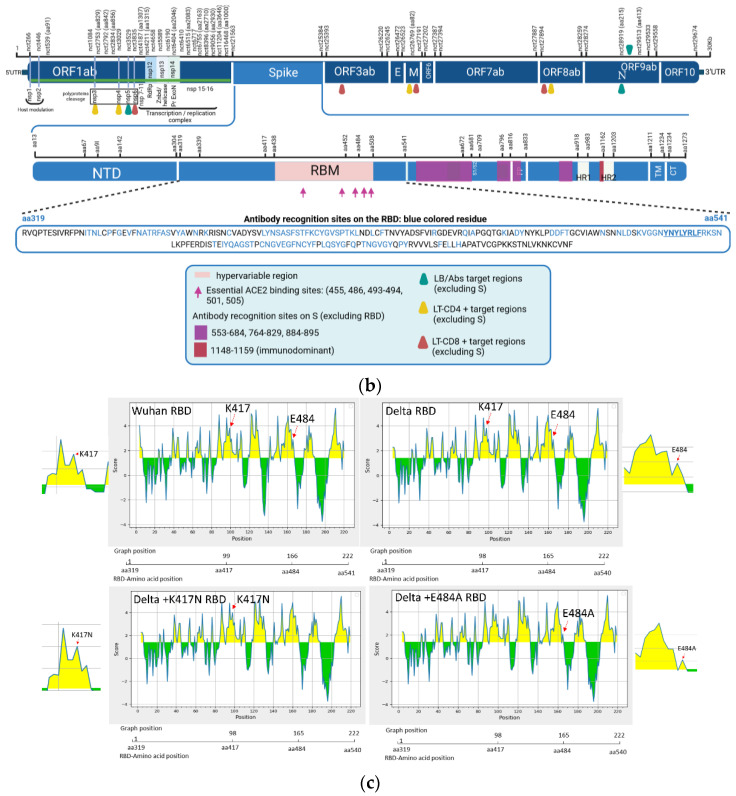
Tracking the Delta Omicron shift and its functional prediction impact. (**a**) SARS-CoV-2 temporal variability (University Hospital-UH Nancy/France) compared to that in France (Santé publique) from October 2021 to April 2022 (curves). Percentage of Omicron and Delta sequences in UH-Nancy from November 2021 up to April 2022 (bars). (**b**) The mapping of some molecular signatures of variant mixtures and/or recombinants from all clinical and WW samples (E1–10 and WW1–WW4), localized mutations on the map; the peptide used in MHC-I binding prediction is underlined in the RBD sequence (EPI ISL 402124) (biorender). aa: amico-acid position; nct: nucleotide position; RBM: receptor binding motif; LB: B Lymphocytes; LT: T Lymphocytes; Abs: Antibodies; S1/S2: proteolytic cleavage site; FP: Fusion Peptide; TM: transmembrane domain; CT: cytoplasmic tail; HR1/HR2: heptad repeat 1 and 2; nsp: non-structural protein; nsp 1–11: pp1a (polyprotein 1a); nsp 1–16: pp1ab (polyprotein 1ab); nsp3: papain-like proteinase; nsp4: membrane spanning protein (with TM2 domain); nsp5: 3C-like protease (3CL) and main protease (Mpro); nsp6: putative transmembrane domain; RdRp: RNA-dependent RNA polymerase; Znbd: zinc binding domain; Pr ExoN: Proofreading Exonuclease; E: envelope; M: membrane; N: nucleocapsid; ORF3ab, 6, 7ab, 8ab, 9ab, 10: accessory proteins (open reading frames). (**c**) Parker hydrophilicity prediction of Delta RBD, Delta with K417N and E484A mutations RBD and Wuhan reference strain RBD. The X-axis represents the amino acid position on the SARS-CoV-2 RBD and the Y-axis represents the hydrophilicity correlated with the immunogenicity (aa position scale is represented below each X-axis); the yellow surface matches to antigenic residues, the green surface matches to non-antigenic residues (IEDB database).

For SARS-CoV-2 monitoring in patients involved in the systematic French national molecular surveillance of the virus, 10% of all screened samples (n = 414) were ultra-deep sequenced from October 2021 to April 2022. Among them, 58% corresponded predominantly to the 21J (Delta) variant, 39% to the 21K (Omicron)/21L (Omicron), and 3 % other variants—20I (Alpha), 19B (A27), or 20A (EU2). From October to December 2021, the Delta variant predominated, with a rate ranging from 91% to 100%, and decreased after that to 37% in January, 2% in February and March 2022, and became undetectable in April 2022. The Omicron variant started to predominate in January 2022 with a percentage of 59% and increased after that to achieve 100% in April 2022 (Figure 1a)—other minor variants were present in November 2021, January, February, and March 2022 with a percentage of 9%, 4%, 2%, and 3%, respectively (Figure 1a).

Samples were analyzed at a mutation threshold of 5% in the S gene. Ten of the patient samples carried a mixture of Delta and Omicron mutation features and these mixtures were then analyzed by exploring the whole genome (depth ranging from 100 to 670 reads/sample) (Table 1). These samples combined molecular features from Delta variants and Omicron/BA.1/BA.2 variants (Table 1). Another Omicron sub-lineage (BA.4) molecular feature was also observed in some mixtures, i.e., samples E1, E2, E6, E9, and E10. Thus, one signature mutation (S142 del) on the ORF1a of this sub-lineage was observed at 6.1% in sample E10 (April 2022) and was already observed at the end of November 2021 in E1 and E2 at 5.1% and 7.6%, respectively. Signature mutations of Omicron or Delta in mixtures were distributed mainly on the S gene and the ORF1ab (Table 1, Figure 1b). For the SARS-CoV-2 sequences, they were performed in the context of the French national network and deposited on the GISAID site. Elsewhere, analyses performed with the Recombination Detection Program on the sequencing data from the 10 samples with SARS-CoV-2 variant mixtures did not highlight recombination events in these samples.

### 3.2. Detection of SARS-CoV-2 Genetic Variability in Raw Wastewater Samples

In the same period, Deepcheck Whole Genome SARS-CoV-2 genotyping was also applied to wastewater (WW) samples (summarized in Table 1). In the samples collected in January, February, and March 2022, the 21K (Omicron)/BA.1 showed proportions of signature mutations, ranging from 38.8% to 43%, against 21L (Omicron)/BA.2, ranging from 2.5% to 17.2%, and 21J (Delta), ranging from 1.7% to 13.8%. Then, the BA.2 replaced the BA.1 in the WW sample from April 2022 with a proportion of 65.5% and contained one signature mutation of 21J (Delta) on the N gene (G215C at 7.8%). The remaining composition matches to 21J (Delta), BA.1/BA.2 (Omicron) specific mutations (32.7–43.4%) that are only present in the sample but not in sequences used as references in the study.

For the main SARS-CoV-2 strains detected in wastewater samples, the Omicron variants were observed as soon as the sampling began from the beginning of January 2022 (Table 1).

Regarding signature mutations, they were distributed throughout the SARS-CoV-2 genome, mainly within ORF1ab and S genes (Table 1).

### 3.3. Mapping of Variants Signatures

A SARS-CoV-2 genome map was designed, to localize some signature mutations from the mixtures, in order to screen for a bioinformatics functional prediction, considering molecular hydrophilic/hydrophobic scale and flexibility, correlated to putative protein antigenicity (Figure 1b,c) [22,23]. The signature mutations of different minor variants in the clinical or WW samples were mainly localized in ORF1ab and the S gene (Figure 1b).

Regarding the Spike, mutations were localized in the N-Terminal Domain (NTD), the receptor-binding domain (RBD) and/or the receptor-binding motif (RBM), S2 domain as well as near the furin cleavage site.

For the ORF1ab area, signature mutations of minor variants were localized throughout the gene on relevant biological regions, such as: the nsp1 and nsp2 (the host cell modulation); between the nsp3 and nsp4 (proteases involved in the cleavage of non-structural viral polyproteins); on the nsp14 (encoding the proofreading exonuclease); and in the nsp15-ns16 (part of the replication/transcription complex) (Table 1, Figure 1b).

### 3.4. Humoral Immunogenicity and MHC-I Binding Affinity Prediction

In order to investigate potential modifications in humoral and cellular immune host-related response according to SARS-CoV-2 mutations, we used bioinformatics tools (IEBD). The Parker hydrophilicity tool was applied to predict the humoral immunogenicity (Figure 1c) of two mutations localized on the RBD (Figure 1b) and considered as molecular signatures, more often observed in the Omicron clade (Table 1). The E484A mutation decreased the hydrophilicity scale by a factor of 2 when present in a Delta variant (Sample E4), in favor of reducing the viral immunogenicity (Figure 1c), while the K417N showed a tendency to increase hydrophilicity correlated to the immunogenicity when it is present in a Delta variant (Sample E3) (Figure 1c).

Moreover, the MHC-I (major histocompatibility complex) binding prediction (NetMHCpan EL 4.1 method, data not shown) was performed on the wild-type Omicron peptide, without or with the L452R mutation (Figure 1b). The prediction allowed us to determine a score correlated to epitope-MHC-I binding affinity for three MHCI alleles (HLA-A*02, HLA-A*03, HLA-A*11); the higher the score, the higher the affinity. Regarding the Omicron wild-type peptide, scores were ranging from 9 to 1.7 × 10^1^ and were higher than those of Omicron peptide carrying the L452R (1 × 10^−3^ to 6.81 × 10^−3^).

## 4. Discussion

SARS-CoV-2 continues to evolve dynamically and variants emerge over time and overwhelm others. According to sanitary authorities (Santé publique, Fr), in France, Omicron started to predominate Delta at the end of December 2021 (W52-12/27/21) and almost disappeared in February 2022 (0.10% Delta vs. 99.90% Omicron) (W9-02/23). In samples collected from patients at the University hospital of Nancy, Omicron (59%) started to predominate Delta (37%) later in January 2021 (Figure 1a). This rapid increase in the Omicron variant over Delta could be associated with a higher transmissibility of Omicron (R0 of 10) compared to that of Delta (R0 of 7) and to a different vaccine efficacy [24]. Furthermore, the ultra-deep wastewater (WW) sequencing confirmed the shift between these two variants and allowed us to follow the SARS-CoV-2 genetic evolution in the whole Nancy population in the same period (January 2022–April 2022) as the investigation of patients samples (Table 1). Wastewater WGS and targeted PCR are relevant to monitor SARS-CoV-2 genetic variability in a large infected population, with both symptomatic and asymptomatic subjects [25].

During this Delta/Omicron shift, sequences carried variant mixtures in 10 patient strains among 414 analyzed samples, by observing different proportions of variants in the same samples linked to the presence of signature mutations of Delta/Omicron variants or different sub-lineages of the same variant (Omicron). Even though no evidence of recombination events was available to us, these molecular signatures were mainly localized in the ORF1ab (539–905 nt; 2753–16,468 nt) and the S (21,764–23,951 nt) genes, next to sites identified in a study as recombination hotspot regions (ORF1ab: 174–2692 nt; S: 21,801–22,281 nt), in a patient who was co-infected with Beta and Delta variants [3].

According to the mapping data (Figure 1b), signature mutations of minor variants were localized near or in regions corresponding to key biological properties for the virus, such as viral protease (nsp3 and nsp4), replicase (nsp15–16, proofreading exonuclease), genes involved in the host cell modulations (nsp1–2), or on the RBD, known until now as a major ACE2 receptor binding region and as the most immunogenic region, as well as next to the furin cleavage site that plays a key role in viral cellular entry.

Concerning the influence of these mutations on immune host-related response, it is a key point of the molecular and biological surveillance organized on SARS-CoV-2 all over the world. As an example, in our study, on the Spike, the hydrophilicity prediction showed that the E484A mutation was in favor of reducing the viral immunogenicity when theoretically present in a Delta variant (Sample E4) (Figure 1c). Moreover, some mutations could lead to cellular immune evasion, which is potentially the case for L452R according to the MHC-I binding prediction results and also the scientific literature [26]. Importantly, the L452R mutation is a crucial signature of recent BA.4 and BA.5 emerging Omicron strains that are now spreading at the beginning of the current summer in France (Santé publique France, July 2022).

Our study was focused on one French University hospital and wastewaters from the same urban area, which could be a limitation. Nevertheless, it relied on systematic and reproducible screening of positive samples for single nucleotide polymorphisms of SARS-CoV-2 variants, according to homogeneous methods. The detection of possible co-infections through multi-centric studies might have been tricky because of possible heterogeneity in variant screening strategies between biology centers. For another limitation, the potential Delta/Omicron co-infections in the 10 patients of the present study could favor recombination events. Even though no possible recombinant was highlighted in our hands, additional methods able to explore longer viral genomic fragments could be informative in the future within the field of variant recombination [5]. At last, complementary analyses, such as neutralizing antibody testing and cellular immune assays against the various variants harboring different mutational patterns, would be informative. Additionally to bioinformatics tools proposed in the present work, such methods on the ability of variants to evade immunity will deserve other immunological studies in the context of various selective pressure applied to viral quasispecies, such as neutralizing monoclonal antibodies [27].

Elsewhere, a promising approach to monitor SARS-CoV-2 spreading has been assumed to be wastewater-based epidemiology (WBE). The exploration of viral load and genetic variability in human samples could be crucial in patients suffering from symptomatic disease, while WBE could allow us to survey the whole infected population, including asymptomatic infected people excreting the virus in feces and, subsequently, in wastewaters. For temporal detection of SARS-CoV-2 strains and variants of concern, the discussion is in progress on the usefulness of wastewater tracing as a predictive tool of SARS-CoV-2 concern and genetic evolution in humans, within vulnerable patients and also in healthy asymptomatic people [8]. As observed in the present work, within wastewater samples, the Omicron variants were observed as soon as the sampling started from the beginning of January 2022, consistently with the increase in the detection of these variants in hospitalized patients. However, in our hands, for variant ultra-deep-sequencing, samples in wastewaters and patients were not frequent enough to know how wastewater monitoring could detect SARS-CoV-2 variant emergence beforehand, when compared to variant tracing in human populations. This tendency was, indeed, reported by previous studies with up to one to two weeks earlier variant emergence before that observed in human samples [25].

## 5. Conclusions

In short, the emergence of the Omicron over the Delta variant was observed between December 2021 and February 2022 in northeast France. The analysis of the wastewater survey of SARS-CoV-2 viral load and genetic variability is in progress, all over the world and in comparison with various infected patients’ populations. At last, the bioinformatics assays and the mapping of some signature mutations from SARS-CoV-2 variant mixtures in spike gene and/or other genomic SARS-CoV-2 key functional regions, such as ORF1ab, suggest a potential acquisition of compensatory functional or surface properties in viruses circulating in humans and in wastewater samples.

## Figures and Tables

**Table 1:** SARS-CoV-2 genome analysis from patient samples (E1–E10) and WW samples (WW1–WW4). The alignment was carried out with Wuhan reference strain (EPI ISL 402124), Delta (EPI ISL 11448607), and Omicron (EPI_ISL_11352845; EPI_ISL_12179603) from GISAID database. For each sample, the following aspects were mentioned: the main assignation clade (NextClade, Pangolin) and, for the other variants features, the signature mutations, their proportion/percentage in the viral population present in the same sample, their localization in the SARS-CoV-2 genome.

Sample	Sampling Date GenBank Number	NexClade/Pangolin Main SARS-CoV-2 Assignation for Each Sample	*Other SARS-CoV-2 Variants*	*Other Variants Features: Signature Mutations*	*Other Variants:* *Mutations Percentage (%)*
E1	Nov-2021(1) ON832655	21J(Delta)/AY.43	BA.1/BA.2 BA.4	ORF1a:E91del S:D796Y ORF7a:S44del ORF1a:S142del	5.6 6.9 5.2 5.1
E2	Nov-2021(2) ON832659	21J(Delta)/AY.122	BA.4 BA.1/BA.2	ORF1a:S142del S:D796Y	7.6 5.2
E3	Nov-2021(3) ON832662	21J(Delta)/AY.125	BA.1/BA.2	S:T95I, K417N	*99* *95.1*
E4	Dec-2021(1) ON832661	21J(Delta)/B1.617.2	BA.1/BA.2	S:G484A	*99.4*
E5	Dec-2021(2) ON832692	21J(Delta)/AY.124	BA.1/BA.2	S:T95I G339D	*97.5* 10
E6	Dec-2021(3) ON832691	21J(Delta)/AY.43	BA.4 BA.1/BA.2	ORF1a:S142del S:D796Y	6.1 16.3
E7	Dec-2021(4) ON832695	21J(Delta)/AY.122	BA.1/BA.2	S:A67V	*99.3*
E8	Dec-2021(5) ON832711	21J(Delta)/AY.43	BA.1/BA.2	S:A67V	*99.4*
E9	Feb-2022 ON832717	21K(Omicron)/BA.x	BA.4	L452R	*72*
E10	Apr-2022 ON832714	21L(Omicron)/BA.2.11	BA.4	ORF1a:S142del S:L452R	6.1 *93.2*
WW1	Jan-2022 ON832058	21K(Omicron)/BA.1	21J(Delta)/BA.2	21J(Delta)-S:L452R,P681R; ORF1a:P2046L BA.2-ORF1a:S135R,T842I	
WW2	Feb-2022 ON832065	21M(Omicron)/n.a.	21J(Delta)/BA.2	21J(Delta)-ORF1b:L829I BA.2-S:S371F,T376A,D405N,R408S; ORF1a-G1307S;ORF1b:R1315C,T2163I	
WW3	Mar-2022 ON832097	21L(Omicron)/n.a.	BA.1/BA.2	BA.1-ORF1a:K856R BA.2-ORF1a:S2083del,L2048I,A2710T	
WW4	Apr-2022 ON832102	21L(Omicron)/BA.2	21J(Delta)	21J(Delta)-N:G215C	

## Data Availability

GISAID site, http://gisaid.org, French national network.
